# Cryopolymerization enables anisotropic polyaniline hybrid hydrogels with superelasticity and highly deformation-tolerant electrochemical energy storage

**DOI:** 10.1038/s41467-019-13959-9

**Published:** 2020-01-07

**Authors:** Le Li, Yu Zhang, Hengyi Lu, Yufeng Wang, Jingsan Xu, Jixin Zhu, Chao Zhang, Tianxi Liu

**Affiliations:** 1 State Key Laboratory for Modification of Chemical Fibers and Polymer Materials, College of Materials Science and Engineering, Innovation Center for Textile Science and Technology, Donghua University, 201620 Shanghai, P. R. China; 2 The Wallace H. Coulter Department of Biomedical Engineering, Georgia Institute of Technology and Emory University, Atlanta, GA 30332 USA; 3 School of Chemistry, Physics and Mechanical Engineering, Queensland University of Technology, Brisbane, QLD 4001 Australia; 4 Shaanxi Institute of Flexible Electronics (SIFE), Northwestern Polytechnical University (NPU), 127 West Youyi Road, 710072 Xi’an, P. R. China; 5 Key Laboratory of Synthetic and Biological Colloids, Ministry of Education, School of Chemical and Material Engineering, Jiangnan University, 214122 Wuxi, P. R. China; 6 Key Laboratory of Materials Processing and Mold (Zhengzhou University), Ministry of Education, Zhengzhou, 450002 P. R. China

**Keywords:** Materials chemistry, Nanocomposites, Energy storage, Gels and hydrogels

## Abstract

The development of energy storage devices that can endure large and complex deformations is central to emerging wearable electronics. Hydrogels made from conducting polymers give rise to a promising integration of high conductivity and versatility in processing. However, the emergence of conducting polymer hydrogels with a desirable network structure cannot be readily achieved using conventional polymerization methods. Here we present a cryopolymerization strategy for preparing an intrinsically stretchable, compressible and bendable anisotropic polyvinyl alcohol/polyaniline hydrogel with a complete recovery of 100% stretching strain, 50% compressing strain and fully bending. Due to its high mechanical strength, superelastic properties and bi-continuous phase structure, the as-obtained anisotropic polyvinyl alcohol/polyaniline hydrogel can work as a stretching/compressing/bending electrode, maintaining its stable output under complex deformations for an all-solid-state supercapacitor. In particular, it achieves an extremely high energy density of 27.5 W h kg^−1^, which is among that of state-of-the-art stretchable supercapacitors.

## Abstract (web summary)

Energy storage devices that can endure large and complex deformations are central to the development of wearable electronics. Here the authors present a cryopolymerization strategy for preparing an anisotropic polyvinyl alcohol/polyaniline hydrogel for flexible supercapacitor electrodes.

## Introduction

Stretchable supercapacitors, enabling desirable portable and wearable electronics applications incapable of using conventional rigid electronics, are highly desirable^1–10^. Although conventional electrodes for supercapacitors are usually not stretchable or superelastic, the engineering of conventional electrodes with geometrically engineered structures provides notable breakthroughs for the achievements of stretchable and foldable devices^11–15^. Nevertheless, geometrically engineered electrodes are incapable of an out-plane bending especially perpendicular to the direction of strain, which is challenging for integrating individual geometrically engineered devices into compact stacking. Besides, most of the geometrically engineered electrodes are challenging to endure both large and complex deformations, substantially limiting their practical applications^16–21^.

Compared to geometrically engineered electrodes, approaches of designing electrode materials with an intrinsic stretchability could impart electrode components and integrated devices with attractive advantages including low-cost and up-scale fabricating features^22^. Among many candidates, conducting polymers are regarded as promising substitutes for electrode materials due to their easy preparation, good conductivity and high theoretical capacitance^23,24^. Hydrogels made from the conducting polymers facilitate a promising combination of high conductivity derived from conducting polymers and excellent stretchability derived from hydrogels.^25–29^ In particular, the hydrogel architecture not only facilitates an efficient electron/ion transport but also suppresses the structural collapse of electrodes by accommodating the volume changes during cycling^19,24,30–33^. However, conducting polymer-based hydrogels still struggle against the mechanical brittleness especially under large and complex deformations. Therefore, the development of conducting polymer-based hydrogels with superelasticity for highly deformation-tolerant supercapacitors is critical yet challenging^34,35^.

Here, we present a cryopolymerization strategy for preparing a highly intrinsically stretchable/compressive/bendable anisotropic polyvinyl alcohol/polyaniline hydrogel (APPH). The as-obtained APPH exhibits a bi-continuous phase structure consisting of ionic conductive polyvinyl alcohol (PVA) and electrochemically active polyaniline (PANI) scaffolds. Benefiting from its high mechanical strength, intrinsically superelastic properties and bi-continuous phase structure with highly efficient electron/ion transports, an all-solid-state supercapacitor (A-SC) with the APPH electrodes exhibits an outstanding specific capacitance of 260 F g^−1^ and 650 mF cm^−2^, remarkably high energy density of 27.5 W h kg^−1^, and excellent stability under the harshly stretching, compressing, and bending operations.

## Results

### Fabrication and structure of hybrid hydrogels

**Fig. 1:**
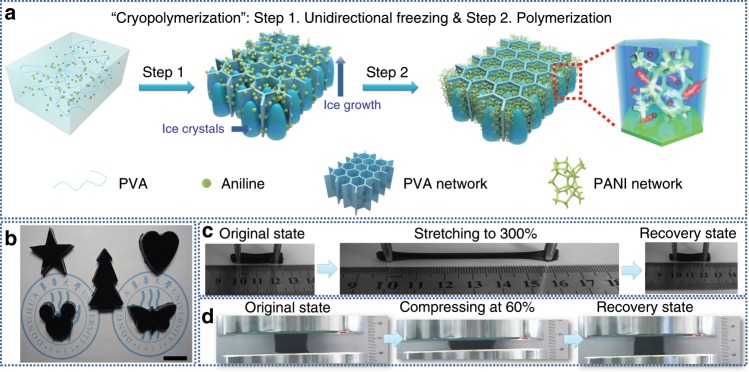
Fabrication and schematic of the anisotropic hybrid hydrogels. **a** Schematic diagram of the preparation procedure of the anisotropic polyvinyl alcohol/polyaniline hydrogel (APPH). **b** Photograph of the APPH with diverse shapes. Scale bar: 2 cm. **c** Photograph of fiber-shaped APPH under stretching/recovering. **d** Photograph of cylinder-shaped APPH under compressing/recovering.

The schematic of the preparation procedure of the APPH is illustrated in Fig. 1a. First, the mixed aqueous solution containing PVA, aniline and initiator was unidirectionally frozen along the vertical-gradient direction, during which a 3D ordered honeycombed structure along the growing direction of ice crystals is formed. Through the subsequent cryopolymerization, a PANI nanofibrous scaffold was gradually formed due to the localized nucleation and confined polymerization of aniline within the boundaries between vertical-aligned ice crystals and PVA cell walls. The polymerization rate of aniline was significantly reduced under the freezing conditions (Supplementary Fig. 1), ensuring that the cryopolymerization of aniline was carried out upon the unidirectional freezing. As a result, the APPH shows an interpenetrating network composed of PVA and PANI scaffolds. The as-obtained APPH is marked as the APPH-1, APPH-2, and APPH-3 when the initial aniline concentration is 0.05, 0.10, and 0.20 M, respectively. A control sample denoted as isotropic PVA/PANI hydrogel (IPPH) was fabricated by randomly freezing with subsequent cryopolymerization, while the other conditions were as same as the preparation of the APPH-2. Another control sample denoted as conventional PVA/PANI hydrogel (CPPH) was prepared by the solution polymerization of 0.10 M aniline on the pre-made PVA anisotropic hydrogel (PVA-AH). However, compared with the APPH-2, unevenly distributed PANI was observed in the CPPH (Supplementary Fig. 2), indicating that the conventional solution polymerization is challenging to achieve a desirable PANI network structure within the pre-made PVA hydrogel scaffold. This can be explained that the early generated PANI nanoparticles would block the further diffusion of aniline into the interior.

Benefiting from the simplicity and easy availability of the cryopolymerization methodology, the APPH with diverse shapes such as stars, hearts, trees, Mickey and butterflies can be readily and precisely fabricated (Fig. 1b). Besides, when subjected to the complex deformations including 300% elongation (Fig. 1c) and 60% compression (Fig. 1d), the APPH can instantaneously recover to its original shape upon the removal of external stress, indicating its outstanding superelasticity. The conductivity of the APPH-1, APPH-2, and APPH-3 is calculated to be 9.5, 19.0 and 20.5 S m^−1^, respectively (Supplementary Fig. 3). These values are much higher than most of the conducting polymer-based hydrogels in the literature (typically in the range of 10^−2^~1 S m^−1^)^36,37^.

**Fig. 2:**
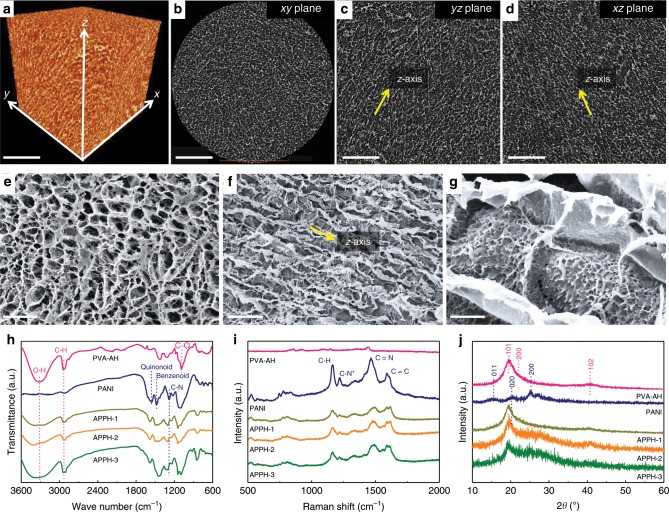
Structural and compositional characterization. **a** Volume rendering of the anisotropic polyvinyl alcohol/polyaniline hydrogel-2 (APPH-2) derived from X-ray computed microtomography (microCT) images, and microCT images display **b** *xy*, **c** *yz,* and **d** *xz* plane, respectively. Scale bars: **a**–**d** 100 μm, respectively. Scanning electron microscopy (SEM) images of the APPH-2 from view of **e** perpendicular and **f**, **g** parallel to *z*-axis. Scale bars: **e**, **f** 20 μm, respectively; **g** 2 μm. Yellow arrow in **f** indicating the *z*-axis. **h** Fourier transform infrared spectra (FTIR), **i** Raman spectra and **j** X-ray diffraction (XRD) patterns of the PANI, PVA anisotropic hydrogel (PVA-AH), and APPH.

During the unidirectional freezing process, the ice crystals preferentially grow along with the vertical-aligned temperature gradient^38–42^, which is hereafter defined as the *z*-axis direction. The plane perpendicular to the *z*-axis is then defined as the *xy* plane. A 3D X-ray micro-computed tomography (microCT) was performed using a Zeiss Xradia 510 Ultra/Versa hybrid system to demonstrate the long-range ordering of the freeze-dried APPH monolith by visualizing its pore features. A 3D volume rendering of the APPH-2 monolith was reconstructed through quantities of the sliced images, suggesting the presence of micron-sized vertical pore structures (Fig. 2a–d)^43^. The APPH samples were also characterized by the scanning electron microscope (SEM) observations. From the view perpendicular to the *z*-axis, tightly packed honeycombed pore structures are observed for the APPH-1 (Supplementary Fig. 4a), APPH-2 (Fig. 2e), and APPH-3 (Supplementary Fig. 4b). The diameter of honeycombed pores gradually decreases with the increase of aniline concentrations. This is due to that the growth of ice crystals in the presence of solutes strongly depends on a balanced adsorption/desorption process of solutes on the growing front of the ice crystals^44^. From the view parallel to the *z*-axis, distinctly periodic structures with the vertical-aligned scaffold are observed for the APPH-1 (Supplementary Fig. 4c, d), APPH-2 (Fig. 2f, g) and APPH-3 (Supplementary Fig. 4e, f). Orientated pore structures are also observed in the PVA-AH (Supplementary Fig. 5). In contrast, the PVA-IH shows a random structure from all directions. During the unidirectional freezing process, the aniline and initiator molecules adsorbing on the solid-liquid interface of ice crystals will hinder the further growth of ice crystals into larger ones. Therefore, smaller pore sizes are observed in the SEM images of the APPH samples with a higher initial aniline concentration of 1.0 M (Supplementary Fig. 6). The formation mechanism of the PANI nanofibrous scaffold was investigated by carrying out the cryopolymerization of aniline and initiator without the addition of PVA. The as-obtained PANI exhibits a unique rod-shaped structure consisting of nanofibrous sub-units (Supplementary Fig. 7). The cryopolymerization is beneficial to the formation of a 3D PANI nanofibrous structure because the closely-packed aniline between the ice crystals will hinder the intermolecular collision of the in-situ growing PANI, compared with the conventional solution-processed polymerization of aniline. For comparison, the CPPH exhibits vertical-aligned PVA scaffolds with the immobilized PANI nanorods of less than 100 nm in the diameter (Supplementary Fig. 8a, b). Although the IPPH maintains the 3D PANI nanofibrous scaffold structure, the pore structures of the PVA scaffold are randomly distributed (Supplementary Fig. 8c, d).

The interfacial interaction between the PANI and PVA scaffolds was further investigated. The presence of PVA and PANI components within the APPH was identified by their typical peaks (Supplementary Table 1) in the Fourier transform infrared (FTIR, Fig. 2h) and Raman spectra (Fig. 2i). The FTIR peaks at 3310 cm^−1^ (O–H stretching vibration) and 1280 cm^−1^ (C–N stretching vibration) among the APPH samples shift to the high wavenumbers, suggesting the formation of O–H^…^N–C hydrogen bonding between the PVA and PANI chains. The Raman peak located at 1450 cm^−1^ (C–N^+^ stretching of semiquinoid rings) is associated with the oxidation of PANI in the APPH. The UV-Vis spectra of the APPH exhibit three prominent absorption bands at 336, 430, and 805 nm (Supplementary Fig. 9), assigned to π-π* transition in benzenoid and two polaron band π-π* transitions in quinoid rings, respectively, which are the features of protonated amine of PANI in its polaron state^45^. Furthermore, the X-ray diffraction (XRD) patterns recorded from the APPH samples exhibit a peak located at 2*θ* = ~21°, corresponding to the (110) plane of the PVA and a series of peaks at 2*θ* = 20–30° assigned to the PANI (Fig. 2j)^44^.

The control sample denoted as PVA isotropic hydrogel (PVA-IH) was prepared by the randomly freezing of a neat PVA solution, to investigate the influence of the freezing methods (randomly and unidirectional freezing) on the crystallinity (*χ*_*c*_) of PVA. The *χ*_*c*_ of PVA-AH and PVA-IH was calculated as 47.3% and 35.6%, respectively, indicating that the unidirectional freezing process effectively improves the molecular regularity of PVA chains (Supplementary Fig. 10). The variation of the crystallinity for the neat PVA and APPH was also proved by the DSC curves (Supplementary Fig. 11) and summarized in Supplementary Table 2. The unidirectional freezing process is conducive to the alignments of PVA chains and folding into the microcrystalline region, leading to the improved crystallinity of PVA among the PVA-AH, which is significantly beneficial for its mechanical enhancement especially along the vertical-aligned direction^41,46^. Besides, the crystallinity of the APPH samples gradually decreases with increased PANI content. Compared with the PVA-AH, the formation of hydrogen bonding between the PVA and PANI among the APPH (proved by FTIR spectra, Fig. 2h) inhibits the formation of PVA microcrystalline. To be noted, the APPH-3 still shows a higher crystallinity than that of the PVA-AH.

### Mechanical performance of hybrid hydrogels

**Fig. 3:**
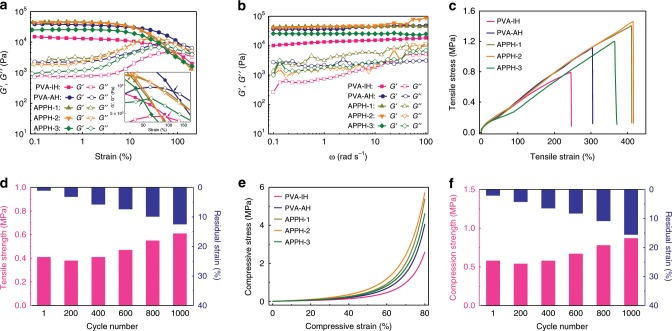
Mechanical performance of the anisotropic hybrid hydrogels. **a** Rheological strain sweeping measurements of PVA anisotropic hydrogel (PVA-AH), PVA isotropic hydrogel (PVA-IH) and anisotropic polyvinyl alcohol/polyaniline hydrogel (APPH) at an angular frequency of 1 rad s^−1^. **b** Angular frequency sweeping measurements of PVA-IH, PVA-AH and APPH at a constant strain of 0.5%. **c** Tensile stress-strain curves of PVA-AH, PVA-IH and APPH. **d** Tensile strength and residual strain of APPH-2 after different stretching cycles of successive loading-unloading at a strain of 100%. **e** Compressive stress-strain curves of PVA-AH, PVA-IH and APPH. **f** Compression strength and residual strain of APPH-2 after different compression cycles of successive loading-unloading at a strain of 50%.

The elastic moduli (G′) of the APPH determined by the rheological measurements are much higher than the corresponding loss moduli (G″) at a small strain (Fig. 3a), indicating that the bulk responses of the APPH to the applied deformation are nearly elastic. The transition points of the APPH (defined by tan δ) are lower than that of the PVA-AH along with an increased strain, which is a signal of a localized viscous behavior with a promoted energy dissipation due to the breakdown of hydrogen bonds between the PVA and PANI^47–49^. The transition strain of the APPH-2 is higher than that of IPPH (Supplementary Fig. 12). Therefore, there exists a robust intermolecular interaction within the APPH-2, verifying that the unidirectional freezing enables the formation of a robust hydrogel network compared with the random freezing. From the sweeping measurements (Fig. 3b), the APPH is ductile due to the incorporation of the stiff PANI network, indicated by their higher G′ and G″ values (10^3^~10^5^ Pa) than that of the PVA-AH (<10^3^ Pa) over the entire frequency range^50^.

Figure 3c indicates that the stiff PANI network simultaneously increases the tensile strength and elongation at break of the APPH compared with those of the PVA-AH and PVA-IH. For instance, the PVA-AH exhibits an elongation at break of 304% with a fracture strength of 1.04 MPa, whereas the elongation at break of the APPH-2 increases to 416%, together with an increased fracture strength of 1.46 MPa. As summarized in Table 1, the APPH-2 possesses superior fracture energy relative to other PVA hydrogels in the literature^18,29,35,51,52^. Besides, the hydrogel with anisotropic pore structures along the deformation direction shows a simultaneously enhanced mechanical strength and elastic modulus, compared with those hydrogels with random pore structures (Supplementary Fig. 13). According to the thermogravimetry analysis (TGA) (Supplementary Fig. 14) and elemental analysis (Supplementary Table 3), the weight contents of PANI in the APPH are determined as 4 wt% (APPH-1), 8 wt% (APPH-2) and 16 wt% (APPH-3), respectively. The influence of the PANI content on the mechanical properties of the APPH was investigated (Supplementary Fig. 15). The tensile strength and elongation at break of the APPH samples considerably increase when the PANI content increases from 1 to 8 wt%. The significant improvement in the tensile strength is ascribed to a unique reinforcing effect of the well-distributed rigid PANI network. The improvement of the elongation at break is related to the destruction of hydrogen bonding interactions between the PANI and PVA networks, as well as the dissociation of the microcrystalline regions, which are helpful for energy dissipations during the stretching. The tensile strength and elongation at break of the APPH samples decrease when further increasing the PANI content to 12 wt% within the APPH. The decrease of the mechanical strength is due to a decreased crystallinity of PVA originating from the excessive loading of PANI, proved by the XRD and DSC results (Supplementary Table 2). Besides, the relatively high loading of PANI among the APPH will lead to a decrease of load transferring from the PVA network to PANI nanostructures, contributing to the decreased elongation at break.

**Table 1:** Summary of mechanical properties of neat polyvinyl alcohol and anisotropic polyvinyl alcohol/polyaniline hydrogels.

Sample	Tensile performance				Compression performance		
	Strength [MPa]	Modulus [MPa]	Elongation at break [%]	Toughness [MJ m^−3^]	Strength [MPa]	Modulus [MPa]	Toughness [MJ m^−3^]
PVA-IH	0.79 ± 0.17	1.80 ± 0.34	247 ± 25	1.12 ± 0.13	2.59 ± 0.21	1.36 ± 0.21	0.30 ± 0.11
PVA-AH	1.04 ± 0.21	3.20 ± 0.53	304 ± 38	1.73 ± 0.34	4.05 ± 0.45	2.65 ± 0.42	0.46 ± 0.05
APPH-1	1.40 ± 0.13	3.85 ± 0.58	411 ± 43	3.18 ± 0.42	5.40 ± 0.72	2.72 ± 0.54	0.62 ± 0.31
APPH-2	1.46 ± 0.34	4.27 ± 0.34	416 ± 46	3.28 ± 0.36	5.74 ± 0.68	2.68 ± 0.42	0.74 ± 0.23
APPH-3	1.20 ± 0.42	3.65 ± 0.67	360 ± 35	2.98 ± 0.43	4.62 ± 0.84	2.98 ± 0.66	0.54 ± 0.28

The superelastic features of the APPH are attributed to the strong interfacial interactions between the PVA and PANI skeletons, as well as the improved crystallinity of the PVA skeletons. The anti-fatigue properties of the APPH-2 were evaluated by carrying out multiple loading/unloading tests (Supplementary Movie 1). Upon 100 tensile/recovery cycles at a 100% strain, the hysteresis loops of the APPH-2 are almost overlapped, indicating its excellent recovering properties (Supplementary Fig. 16a). The tensile strength of the APPH-2 maintains 100% of its original value after 400 loading/unloading stretching cycles (Fig. 3d). The tensile strength of the APPH-2 slightly increases, especially after long-term loading/unloading stretching cycles, which is ascribed to the mechanical enhancement caused by inevitable water evaporations during the fatigue tests. Despite 16.5% of the residual strain remains after 1000 successive stretching cycles, the residual strain of the APPH-2 reduces to only 0.5% after the recovery for 2 h, showing its excellent recovery performance (Supplementary Fig. 17). Furthermore, the APPH-2 exhibits excellent compression stability under an 80% strain, which is more than 41% improvement compared with that of the PVA-AH (Fig. 3e). Furthermore, the anti-fatigue tests of the APPH-2 indicate its excellent compression/recovery performance under the repeated compression cycles at a 50% strain (Fig. 3f, Supplementary Fig. 16b, Supplementary Movie 2).

**Fig. 4:**
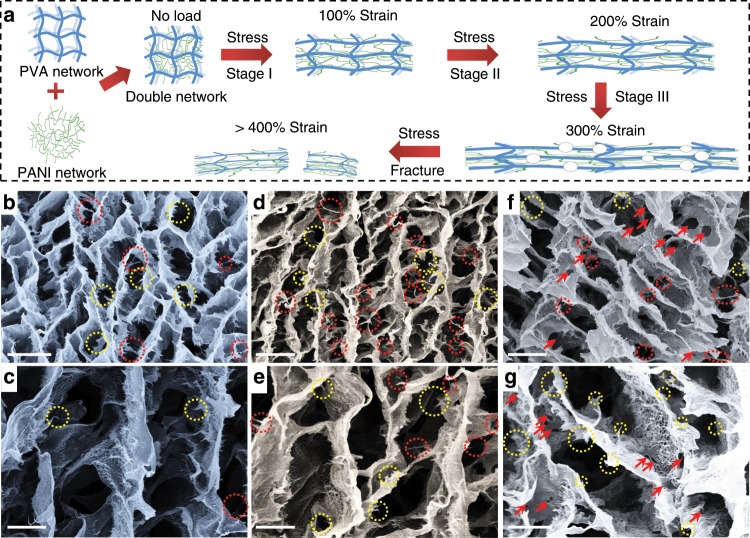
Strengthening and toughening mechanism. **a** Schematic illustration of the stretching process of the anisotropic polyvinyl alcohol/polyaniline hydrogel-2 (APPH-2). Ex-situ scanning electron microscopy (SEM) observations of the APPH-2 stretched at the strain of **b**, **c** 100%, **d**, **e** 200%, and **f**, **g** 300%. Scale bars: **b**, **d**, **f** 10 μm, respectively; **c**, **e**, **g** 4 μm, respectively.

The strengthening and toughening mechanism for the APPH were further studied. The PANI sacrificial network, entangled with the PVA network through dynamic hydrogen bonding (proved by FTIR results in Fig. 2h), significantly increases the crosslinking density of the APPH with a unique double network (Fig. 4a). When an external loading is applied (i.e., 100% strain at Stage I), the facture energy is efficiently dissipated due to the deformation of vertical-aligned pores among the APPH. In this case, most of the applied loadings are primarily transferred to the stiff PANI network (ex-situ SEM images, Fig. 4b, c)^53,54^. At the same time, the PANI network entangled with the PVA chains bridging neighboring vertical-aligned pores can be pulled out to dissipate more energy (indicated by the red circles, Fig. 4b, c). When the loading increases (i.e., 200% strain at Stage II), more disruptions of the PANI network bridging adjacent PVA scaffolds are observed (indicated by the yellow circles, Fig. 4d, e). When further increasing the loading (i.e., 300% strain at Stage III), micro-cracks and holes are formed for the dissociations of the microcrystalline regions and physical entanglements of the PVA chains (indicated by the red arrows, Fig. 4f, g), effectively consuming the energy under the large deformations and thus resulting in the extremely high fracture energy of the APPH^40^. The energy-dissipating processes under the different strain conditions effectively reorganize the double-networked composite hydrogels and distribute the applied loading rapidly and uniformly over the entire sample^47^. By contrast, the PVA-IH with the randomly coiled polymer chains is neither highly stretchable nor compressible, since it only dissipates energy through the ruptures of entangled PVA chains. Therefore, the improvement of the mechanical strength of the APPH is due to the increased crosslinking density ascribing to the formation of the rigid PANI network and microcrystalline regions of PVA. The toughening mechanism is contributed to the formation of the effective energy dissipation pathways, due to the destruction and reversible reconstruction of interfacial interactions between the PVA and PANI phases, as well as the sacrificial fracture of the rigid PANI network and the dissociation of the microcrystalline region of PVA^18,49,55–57^. Benefiting from the robust double-network structure of the highly interconnected PANI, the APPH-2 guarantees a fast electron transport, which maintains nearly 90% of its conductivity even under large deformations, such as 200% stretching, 50% compression and 180° bending (Supplementary Fig. 18).

### Electrochemical performance of hybrid hydrogels

**Fig. 5:**
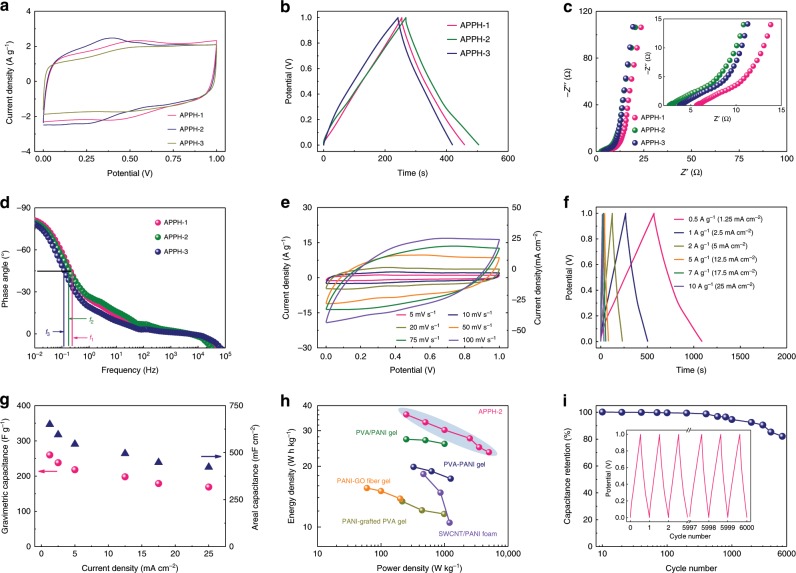
Electrochemical performance of the anisotropic hydrogel electrodes. **a** Cyclic voltammetry (CV) curves of the all-solid-state supercapacitor (A-SC) with anisotropic polyvinyl alcohol/polyaniline hydrogel-1 (APPH-1), anisotropic polyvinyl alcohol/polyaniline hydrogel-2 (APPH-2) and anisotropic polyvinyl alcohol/polyaniline hydrogel-3 (APPH-3) electrodes, respectively, at a scan rate of 10 mV s^−1^. **b** Galvanostatic charge/discharge (GCD) curves of the A-SC with APPH-1, APPH-2 and APPH-3 electrodes, respectively, at a current density of 1 A g^−1^. **c** Nyquist plots of the A-SC with APPH-1, APPH-2 and APPH-3 electrodes, respectively. Inset of **c** showing high-frequency region of Nyquist plots. **d** Bode plots of the A-SC with APPH-1, APPH-2 and APPH-3 electrodes, respectively. **e** CV curves of the A-SC with APPH-2 electrodes at different scan rates. **f** GCD curves of the A-SC using APPH-2 electrodes at different current densities. **g** Gravimetric and areal capacitances of the A-SC with APPH-2 electrodes at different current densities. **h** Ragone plots of the A-SC with APPH-2 electrodes in comparison to other reported supercapacitors with 3D conducting polymer electrodes. **i** Cycling performance of the A-SC with APPH-2 electrodes for 6000 cycles. Inset of **i** showing GCD curves during the first and last three cycles.

The electrochemical properties of the APPH as electrodes for the A-SC were evaluated by cyclic voltammetry (CV) and galvanostatic charge/discharge (GCD) tests. The surrounded areas of the CV curves of the APPH electrodes at 10 mV s^−1^ demonstrate their capacitances following the order of APPH-3 < APPH-1 < APPH-2 (Fig. 5a, Supplementary Fig. 19), which is consistent with the results based on the GCD tests (Fig. 5b). Nyquist plots of the A-SC with the APPH electrodes show the high-, mid-, and low-frequency regions, corresponding to the electro-transfer limited process, diffusion-limited electrode process and capacitive behavior (Fig. 5c)^58^, respectively. In the mid-frequency region, the slope of Nyquist plots represents the Warburg impedance (*Z*_*w*_), indicating the electrolyte diffusion within the electrode^59,60^. The calculated *Z*_*w*_ value of the APPH-3 from the fitted equivalent circuit (Supplementary Fig. 20) is higher than those of the APPH-1 and APPH-2 (Supplementary Table 4), implying the slightly sluggish ion diffusion kinetics among the APPH-3 because the excessive PANI particles would block the ion diffusions through the vertical-aligned scaffold. This result is also reflected by the largest *RC* time constant (8.33 s) of the APPH-3 among the APPH samples in the corresponding Bode plots (Fig. 5d). At the high-frequency region, the charge-transfer behavior at the electrode–electrolyte interface is expressed as a semicircle. The appearance of the distorted semicircular arc in Fig. 5c results from the overlapping of the high-frequency redox and mid-frequency reactions due to the presence of a 3D interconnected PANI nanofibrous scaffold^61^. Besides, the intersection point on the real axis shows the equivalent series resistance (*R*_*s*_) of the electrochemical system, which is a combination of the solution resistance, intrinsic resistance of electrodes and interfacial resistance between the electrode and current collector^62^. The A-SC with the APPH-2 electrodes shows an efficient charge transfer process, proved by the smallest *R*_*s*_ and largest contact impedance (defined as *C*_*1*_*/R*_*ct*_) (Supplementary Table 4). The excellent charge transfer and fast ion diffusion therefore enable the APPH-2 electrode with optimized capacitive performance among the APPH electrodes. The CV curves of the A-SC with the APPH-2 electrodes exhibit the redox peaks at different scan rates, indicating its dominated pseudocapacitive characteristics based on the redox mechanism (Fig. 5e). The gravimetric and areal capacitances of the A-SC with the APPH-2 electrodes at different current densities are calculated based on the GCD curves (Fig. 5f). The A-SC with the APPH-2 electrodes delivers a high gravimetric and areal capacitance of 260 F g^−1^ and 650 mF cm^−2^, respectively, at a current density of 0.5 A g^−1^, which are much higher than those of flexible supercapacitors with conducting polymer-based hydrogel electrodes in the literature (Supplementary Table 5). When the discharging current density of the A-SC with the APPH-2 electrodes increases from 0.5 to 5 A g^−1^, its gravimetric and areal capacitances are still well retained at 198 F g^−1^ and 495 mF cm^−2^ (Fig. 5g), respectively, revealing its excellent rate capabilities. The electrochemical performance of the A-SC with the APPH-2 electrodes of different thicknesses is investigated (Supplementary Fig. 21). The gravimetric capacitance of the A-SC is well maintained with only a small decrease, implying that the continuous PANI phase of 3D interconnected nanofibrous scaffold endows a fast electron transport and ion diffusion in the APPH-2 matrix. In contrast, an increase in the thickness of conventional electrodes will inevitably lead to the reduction of the electron/ion transferring efficiency and subsequent gravimetric capacitance performance. Figure 5h demonstrates the Ragone plots of the A-SC with the APPH-2 electrodes in comparison to the state-of-the-art flexible supercapacitors with 3D conducting polymer-based electrodes. The A-SC with the APPH-2 electrodes exhibits a high energy density of 27.5 W h kg^−1^ at a power density of 5.0 kW kg^−1^, which is among the top of the-state-of-the-art stretchable supercapacitors with conducting polymer-based electrodes^18,19,26,51,52^. Besides, the APPH-2 electrode also presents excellent cycling stability with more than 90% and 80% of its original specific capacitance retained after 2000 and 6000 charge/discharge cycles, respectively (Fig. 5i). Conducting polymer-based electrodes typically suffer from a rapidly decayed capacitance, which is due to the inevitable volume changes during the cycling^63^. The APPH electrodes with a 3D interconnected PANI nanofibrous scaffold provide an efficient charge transport, short ion diffusion pathway and 3D porous feature accommodating the volume changes. Also, the APPH containing the bi-continuous ionic conductive/electrochemically active phases contributes to the molecular-level contacting of PANI phases with the electrolytic ions, resulting in an extremely high electrochemically active surface area. These unique structural characteristics simultaneously make contributions to the promotion of the as-fabricated A-SC with largely enhanced energy storage performance and cycling stability.

**Fig. 6:**
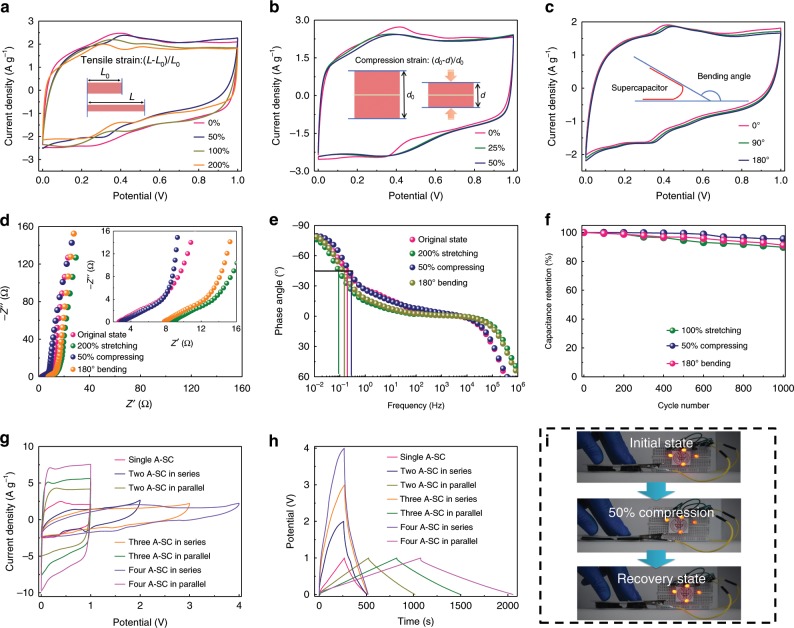
Performance of all-solid-state supercapacitors under large/complex deformation. Cyclic voltammetry (CV) curves of the all-solid-state supercapacitor (A-SC) under **a** stretching, **b** compressing and **c** bending. Insets in **a**, **b**, **c** demonstrating the definition of tensile strain, compression strain and bending angle, respectively. **d** Nyquist plots of the A-SC under different deformation states, respectively. Inset of **d** showing magnified high-frequency region of Nyquist plots. **e** Bode plots of A-SC under 200% stretching, 50% compression and 180° bending respectively. **f** Capacitance retention of the A-SC after repeated 100% stretching, 50% compression and 180° bending cycles, respectively. **g** CV and **h** Galvanostatic charge/discharge (GCD) curves of one, two, three and four A-SC connected in series and parallel at 10 mV s^−1^ and 1 A g^−1^, respectively. **i** Photographs showing an integrated device with four series-connected A-SC lightening up 4 light-emitting diodes (LEDs) under compressing/recovering.

The as-fabricated A-SC with the APPH-2 electrodes is highly stretchable, compressible and bendable due to the intrinsically strong and ductile mechanical properties of the APPH electrodes. The A-SC exhibits high retentions of gravimetric capacitances under various deformations (e.g., stretching, compression and bending at different degrees), as demonstrated by their almost overlapped CV curves in Fig. 6a–c, respectively. Its gravimetric capacitances calculated from the GCD curves (Supplementary Fig. 22), achieve more than 85% of its original capacitance when stretched to 200% or bent at 180°. Under a 50% compression, the A-SC can maintain nearly 100% of its original capacitance. In addition, its electron transport and ion diffusion under various deformations were investigated by carrying out the EIS measurements in the form of Nyquist and Bode plots (Fig. 6d, e). Supplementary Fig. 20 indicates the fitted equivalent circuit of the A-SC, and the fitting parameters are summarized in Supplementary Table 6. Increased equivalent series resistance (*R*_*s*_) and contact impedance (*C*_*1*_*/R*_*ct*_) values are observed when the A-SC is stretched to 200% or bent to 180°, implying the slightly deteriorated electron transport and thus decreased capacitances during the deformations. The fast *RC* time constant is observed for the A-SC under a 50% compression, indicating that the 3D interconnected PANI nanofibrous scaffold provides extremely stable charge and ion transports. As a result, the as-fabricated A-SC shows no apparent deteriorated electrochemical performance after the repeated stretching, compressing and bending cycles (Fig. 6f).

Assembly of individual supercapacitors in series and parallel circuits is an effective way to explicitly tailoring the output voltage and discharging capacity. Here, the assembly of two, three and four series- and parallel-connected A-SC with APPH-2 electrodes is demonstrated with enlarged output voltage and prolonged discharging time (Fig. 6g, h). For instance, the highest output voltage of four devices in series is four-fold of the single device at the same current density, and the discharge time of the four devices in parallel is four-fold of the single device. As a proof-of-concept, an integrated supercapacitor device was assembled with four series-connected A-SC. Supplementary Fig. 23 shows the rational design of an integrated unit, in which Au film is patterned on a PDMS film to guarantee the flexibility of the whole device. The integrated supercapacitor device can simultaneously light up four light-emitting diodes (LEDs) under a significant deformation of 50% strain (Fig. 6i). Also, it can provide high enough discharging capacity to lighten the LEDs for several minutes (real-time photographs in Supplementary Fig. 24), demonstrating its extraordinary energy storage capability.

### Contribution of microstructure on electrochemical property

**Fig. 7:**
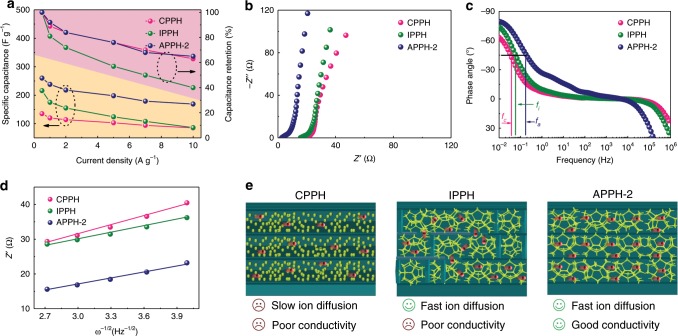
Contribution of microstructure on electrochemical properties. **a** Specific capacitance and capacitance retention of the conventional PVA/PANI hydrogel (CPPH), isotropic PVA/PANI hydrogel (IPPH) and anisotropic polyvinyl alcohol/polyaniline hydrogel-2 (APPH-2) electrodes. **b** Nyquist and **c** Bode plots of the CPPH, IPPH and APPH-2 electrodes, respectively. **d**) Linear fitting showing relationship between *Z*′ and *ω*^*−*1/2^ of the CPPH, IPPH and APPH-2 electrodes. **e** Schematic illustration of the contribution of the microstructure on the ion and electron tranport properties of the CPPH, IPPH and APPH-2 electrodes.

The influence of the microstructure on the electrochemical performance was investigated by comparing the APPH-2, CPPH, and IPPH electrodes. As illustrated in Fig. 7a, the APPH-2 electrode exhibits largely enhanced specific capacitances and improved rate capabilities compared with that of CPPH and IPPH electrodes, especially at large current densities. The corresponding EIS curves (Fig. 7b) and Bode plots (Fig. 7c) indicate that the APPH-2 possesses optimized charge transfer and ion diffusion capacity, as proved by the smallest *R*_*s*_ and *RC* time constant, respectively. From the relationship between *Z*′ and *ω* (*ω* = *2π* *×* *f*) in the low-frequency region (Fig. 7d), the APPH-2 electrode presents the lowest slope among the three electrodes, demonstrating its optimized ion diffusion kinetics. In contrast, the IPPH with randomly distributed pore structure is unfavorable for rapid charge transfer because the disordered microchannels prolong electron transfer pathways, which is also reflected in the largely improved *R*_*s*_ in the EIS curve of IPPH (Fig. 7e). The inferior charge transfer and ion diffusion are demonstrated for the CPPH due to its largest *R*_*s*_ (Fig. 7b) and fitting slope of *Z*′ and *ω* (Fig. 7d). Uneven distributed PANI with aggregations among the CPPH goes against the effective electron and ion transfer between isolated PANI particles (Fig. 7e). In order to quantitatively verify this hypothesis, the apparent diffusion coefficients (*D*_*0*_) of electrolytic ions in the electrodes were calculated according to the Randles-Sevcik equation (Eq. 1):

1 $$i_p = (2.69 \times 10^5)n^{\frac{3}{2}}{{AD}_0}^{\frac{1}{2}}C_0^ \ast v^{\frac{1}{2}}$$

Where *i*_*p*_ is the peak current, *n* is the electron transfer number, *A* is the electrode surface area, the *D*_*0*_ is the diffusion coefficient, $C_0^ \ast$ is the reactant concentration, and *v* is the scan rate. The diffusion coefficients of the APPH-2 (*D*_APPH-2_) and IPPH (*D*_CPPH_) are calculated according to the CV curves (Supplementary Fig. 25) and the following formula (Eq. 2).

2 $$D_{{\mathrm{APPH}} - 2}/D_{{\mathrm{CPPH}}} = \left[ {\left( {i_p/v^{1/2}} \right)_{{\mathrm{APPH}} - 2}/\left( {i_p/v^{1/2}} \right)_{{\mathrm{CPPH}}}} \right]^2 = 2.92$$

The *D*_APPH-2_ is determined to be 0.035 cm^2^ s^−1^, which is 2.92 times larger than that of *D*_CPPH_ (0.012 cm^2^ s^−1^), indicating that highly interconnected PANI structure among the APPH is beneficial for the efficient ion diffusion.

Owing to the anisotropic porous structure with a 3D conductive PANI framework, the APPH-2 electrode exhibits largely enhanced specific capacitances compared with that of the CPPH and IPPH, especially at the relatively large current densities. Although the APPH-2 shows the vertical-aligned porous structure, the electrochemical performance of the APPH-2 shows no differences when considering the placement direction of the electrodes (Supplementary Fig. 26). This finding is of significant importance of the anisotropic porous electrodes in the practical applications.

## Discussion

We present a vertical-gradient freezing and cryopolymerization strategy for designing and preparing an anisotropic APPH. During the vertical-gradient freezing, the PVA chains, aniline monomer and initiator could assembly into a 3D ordered honeycombed structure due to the oriented growth of ice dendrites. Through the subsequent cryopolymerization, the PANI nanofibrous scaffold was gradually formed due to the localized nucleation and confined polymerization of aniline within the boundaries between vertical-aligned ice crystals and PVA cell walls. The cryopolymerization method is easily extended to the preparation of other conducting polymer-based hydrogels, i.e., anisotropic PVA/polypyrrole (PPy) hydrogel. The as-prepared anisotropic PVA/PPy hydrogel also exhibits impressive superelastic property and unique bi-continuous phase structure (Supplementary Fig. 27).

The as-prepared APPH shows the superelastic performance with a complete recovery of 100% stretching strain, 50% compressing strain and fully bending, which is due to the effective energy dissipations of the reversible deformation of the PVA scaffold network and PANI sacrificial network, as well as the efficient load transfer ascribed to the strong interfacial interaction between the ductile PVA and stiff PANI networks. The PVA phase with an improved crystallinity induced by the unidirectional freezing process also contributes to the mechanical enhancement of the APPH. In addition, the APPH exhibits bi-continuous ionic conductive/electrochemically active phases. Namely, the 3D PANI nanofibrous scaffold phase provides abundant electrochemically active sites, and the PVA scaffold phase facilitates an efficient ion diffusion. As a result, the APPH exhibits supreme electrochemical performance as an intrinsically stretchable, compressible and bendable electrode for a complex deformation-tolerant A-SC. The as-fabricated A-SC with the APPH-2 electrodes delivers a remarkably high specific capacitance (260 F g^−1^ and 650 mF cm^−2^ at 0.5 A g^−1^) and large energy density (27.5 W h kg^−1^), which is among the top of the state-of-the-art stretchable supercapacitors with conducting polymer-based electrodes. Our studies might pave the way to the development of robust conducting polymer hybrid hydrogel electrodes for highly deformation-tolerant energy storage applications.

## Methods

### Materials

PVA (*M*_*w*_ = 85000–124000, 99+% hydrolyzed) and aniline (ACS reagent, ≥99.5%) were obtained from Sigma-Aldrich. Ammonium persulfate (APS, ACS reagent, ≥98.0%) and hydrochloric acid (CP, 36–38%) were purchased from Sinopharm Chemicals. Deionized (DI) water was used throughout the experiments.

### Preparation of neat polyvinyl alcohol hydrogels

The PVA solution was prepared by dissolving the PVA pellets in DI water at 95 °C under stirring. The as-prepared PVA solution was added into a plastic tube, where a cylindrical copper billet was placed at the bottom. The PVA-AH was obtained by dipping the plastic tube into liquid nitrogen at a rate of 5 mm min^−1^, setting at −5 °C for 72 h and thawing. The PVA-IH was prepared by rapidly immersing the plastic tube into liquid nitrogen, while the other conditions are as same as the preparation of the PVA-AH. The shapes of the PVA-AH and PVA-IH were tailored by employing various plastic molds.

### Preparation of hybrid hydrogels

Designed amounts of aniline, APS and HCl were added into the PVA solution under stirring at 0 °C (Solution A). Subsequently, Solution A was quickly transferred to a plastic tube, where a cylindrical copper billet was placed at the bottom. The plastic tube was dipped slowly into liquid nitrogen at a rate of 5 mm min^−1^. The frozen sample was maintained at −5 °C for 72 h realizing the cryopolymerization of aniline. Upon thawing, the APPH samples were immersed into excess water to remove the impurities and oligomers. The as-obtained APPH is marked as the APPH-1, APPH-2 and APPH-3 when an initial aniline concentration of 0.05, 0.10, and 0.20 M is used, respectively, with the addition of an equal molar amount of APS. The concentration of the PVA is kept at 12 wt%, while the concentration of HCl is kept at 1 M. The IPPH was prepared by rapidly immersing Solution A into liquid nitrogen with a subsequent cryopolymerization, while the other conditions are as same as the preparation of the APPH-2. The CPPH was prepared by in-situ solution-processed polymerization of aniline within the PVA-AH, while the other conditions are as same as the preparation of the APPH-2.

### Materials characterization

The morphologies of hydrogel samples were examined by SEM (Ultra 55, Zeiss) at an acceleration voltage of 5 kV. Porosity and 3D macrostructure analyses were performed in a Zeiss Xradia 510 Versa 3D X-ray microscope (XRM), with a voltage of 60 kV and a power of 10 W. The resulting images were reconstructed by Carl Zeiss’s Object Research Systems (ORS) image analysis program. The chemical compositions of freeze-dried hydrogel samples were analyzed by Fourier transform infrared (FTIR), UV-vis, wide-angle X-ray diffraction (XRD), differential scanning calorimetry (DSC) and Raman spectra. FTIR spectra were measured using freeze-dried hydrogel samples with a Nicolet 570 infrared spectrophotometer (Nicolet 570, USA) at a 4 cm^−1^ resolution, with 64 scans at a scan range of 500–3500 cm^−1^. UV-vis spectra were recorded on a UV-vis spectrophotometer (TU-1810, Beijing Pushi). XRD analyses were performed on Dutch Philips X-ray DY-1291 diffractometer with Ni filtered Cu K_α_ (40 kV, 35 mA) at 2*θ* of 2–60°. DSC experiments were carried out on a NETZSCH TA-204F1 instrument in nitrogen. Raman spectra were measured on a Dilor LABRAM-1B multi-channel confocal microspectrometer with a 631 nm laser excitation. The weight contents of PANI within freeze-dried APPH were investigated by thermogravimetry (TGA) on a thermo analyzer (TG209F1, NETZSCH, USA) and elemental analyses based on a combustion method on elementar (VarioEL-III, Germany). The mechanical properties of hydrogel samples were measured using an Instron Universal Testing Machine (Model 5567). For tensile measurements, fiber-shaped hydrogel samples (molded species with a diameter of 5 mm and length of 3 cm) were stretched at a strain rate of 10 mm min^−1^. For compression tests, cylinder-shaped hydrogel samples set on the lower plate were compressed by the upper plate at a strain rate of 5 mm min^−1^. Rheological measurements were performed on a rheometer (Anton Paar MCR302, Austria). Strain sweeping measurements were conducted at a strain from 0.1 to 200% at a constant angular frequency of 1 rad s^−1^, and angular frequency sweeping was performed from 0.1 to 100 rad s^−1^ at a constant strain of 0.5%.

Calculation of crystallinity (*χ*_*c*_): the *χ*_*c*_ is calculated from XRD patterns and DSC curves, respectively.

The *χ*_*c1*_, obtained from XRD patterns through an interactive peak-fit procedure, can be estimated by Eq. 3:

3 $$\chi _{c1} = \frac{{\Sigma A_{{\mathrm{crys}}}}}{{\Sigma A_{{\mathrm{crys}}} + \Sigma A_{{\mathrm{amor}}}}}$$

Where *A*_*crys*_ and *A*_*amor*_ are the fitted areas of crystals and amorphous phases.

The *χ*_*c2*_ obtained from DSC curves can be calculated by Eq. 4:

4 $$\chi _{c2} = \frac{{\Delta H_m}}{{\Delta H_0}}$$

Where *ΔH*_*0*_ is enthalpy of pure PVA crystals (138.6 J g^−1^)^64^, and *ΔH*_*m*_ is melting enthalpy measured by DSC.

### Electrochemical measurement

Conductivity measurement of APPH: The as-prepared APPH with a fixed shape (length: 2 cm; width: 1 cm; thickness: 1 cm) was sandwiched between two pieces of stainless steel and connected to a CHI 660E workstation to measure their electrical conductivity.

The conductivity of APPH samples was determined by EIS measurements and calculated by Eq. 5:

5 $$\sigma = L/\left( {R \times S} \right)$$

where *L*, *S*, and *R* are the height, area and bulk resistance of hydrogel samples, respectively. *R* is the bulk resistance at the frequency where the phase angle approaches zero in Bode plots.

Fabrication of A-SC: A PVA/H_2_SO_4_ gel, prepared by adding 1 g PVA in 10 mL of 1 M H_2_SO_4_ at 90 °C under vigorous stirring, was used as the solid-state electrolyte. As-prepared APPH were soaked in 1 M H_2_SO_4_ for 6 h, coated with a layer of freshly made PVA/H_2_SO_4_ gel and then dried at room temperature for 0.5 h. Stretchable and bendable A-SC was then assembled by laminating two rectangle-shaped APPH samples (length: 2 cm, width: 1 cm, thickness: 0.2 cm) together, while compressible A-SC was assembled by laminating two cylinder-shaped APPH samples (diameter: 2 cm, thickness: 0.2 cm) together.

The electrochemical performance of A-SC was evaluated in a two-electrode system with a CHI 660E workstation. CV tests were performed in the potential range of 0 to 1 V under a scan rate of 5–100 mV s^−1^. GCD tests were performed by scanning from 0 to 1 V at the current density from 0.5 to 10 A g^−1^. EIS measurements were conducted with a frequency range of 10^5^ Hz to 0.01 Hz with a 10 mV amplitude at open circuit potential. Cycling stability of electrodes was carried out by repeating the GCD tests at 1 A g^−1^. Deformation-tolerant performance of A-SC was evaluated by measuring the CV and GCD curves when A-SC device was stretched, compressed and folded at different degrees. Gravimetric capacitances of A-SC were calculated from GCD curves according to Eq. 6:

6 $$C_s = I \times t/\left( {M \times V} \right)$$

where *I* is the discharge current; *t* is the discharge time; *M* is the total mass of PANI within two electrodes; and *V* is the voltage change upon discharging.

Energy density and power density of A-SC were obtained based on Eqs. 7 and 8, respectively:

7 $$E = \left( {1/2} \right) \times C_s \times V^2$$

8 $$P = E/t$$

where *E*, *C*_*s*_, *V*, *P*, and *t* are the energy density, gravimetric capacitance of A-SC, voltage change upon discharging, power density and discharge time, respectively.

## Supplementary information

Supplementary information

Description of additional supplementary files

Supplementary Movie 1

Supplementary Movie 2

## Data Availability

The data that support the findings of this study are available from the corresponding author upon reasonable request.
