# Supplementary material for: Cryopolymerization enables anisotropic polyaniline hybrid hydrogels with superelasticity and highly deformation-tolerant electrochemical energy storage
Source: Nat Commun. 2020 Jan 7;11:62. doi: 10.1038/s41467-019-13959-9 (PMC6946679; doi:10.1038/s41467-019-13959-9)
Supplement: Supplementary file 1 — Supplementary information [file 41467_2019_13959_MOESM1_ESM.pdf]

Supplementary Information

Cryopolymerization enables anisotropic polyaniline hybrid hydrogels with superelasticity and highly deformation-tolerant electrochemical energy storage

Li et al.

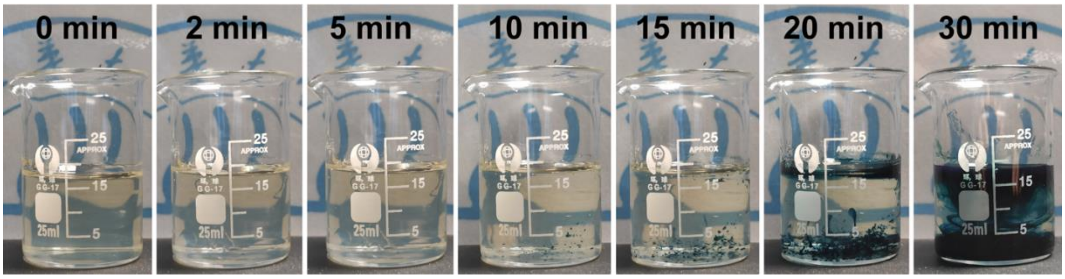

Supplementary Figure 1. Real-time photographs showing the color change of the precursor solution of APPH-2 at 0 ^{o}C during 0 to 30 min.

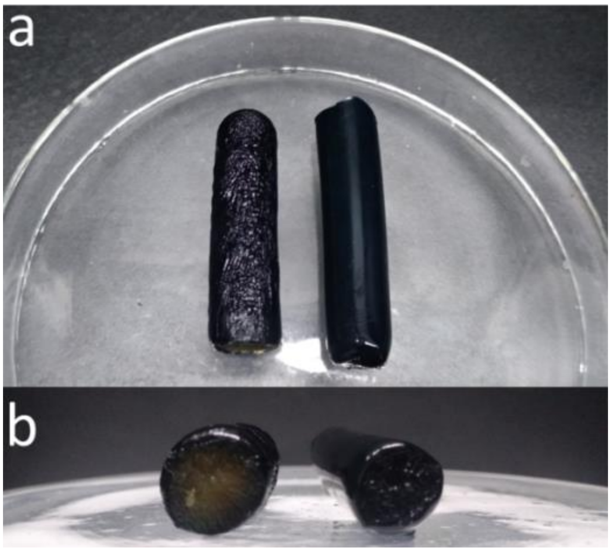

Supplementary Figure 2. Photographs of the CPPH (left) and APPH-2 (right) cylinders from the **a** top and **b** cross-sectional views.

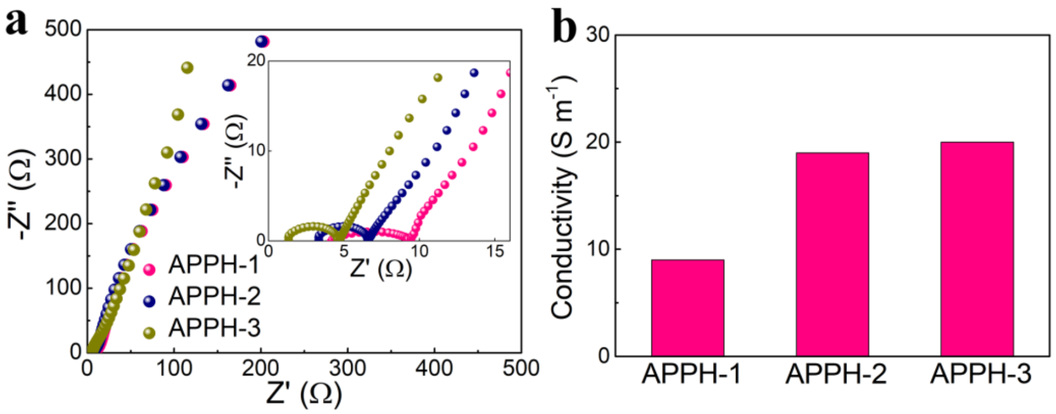

Supplementary Figure 3. a Nyquist plots of APPH-1, APPH-2 and APPH-3 electrodes, respectively. Inset of **a** shows their high-frequency regions of Nyquist plots. **b** Conductivity of APPH-1, APPH-2 and APPH-3 calculated from the EIS measurements.

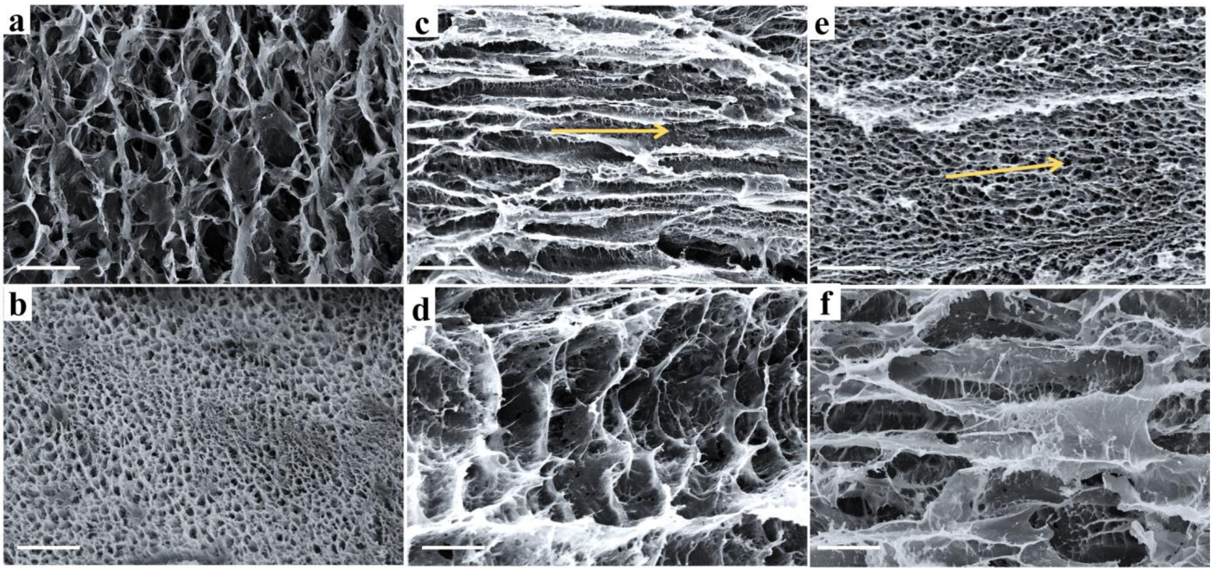

Supplementary Figure 4. SEM images of **a** APPH-1 and **b** APPH-3 from the view perpendicular to the *z*-axis. Scale bars: **a**, **b** 20 μm, respectively. SEM images of **c**, **d** APPH-1 and **e**, **f** APPH-3 from the view parallel to the *z*-axis. Yellow arrows in **c**, **e** indicating the *z*-axis. Scale bars: **c** 20 μm, **d** 2 μm, **e** 20 μm, **f** 2 μm.

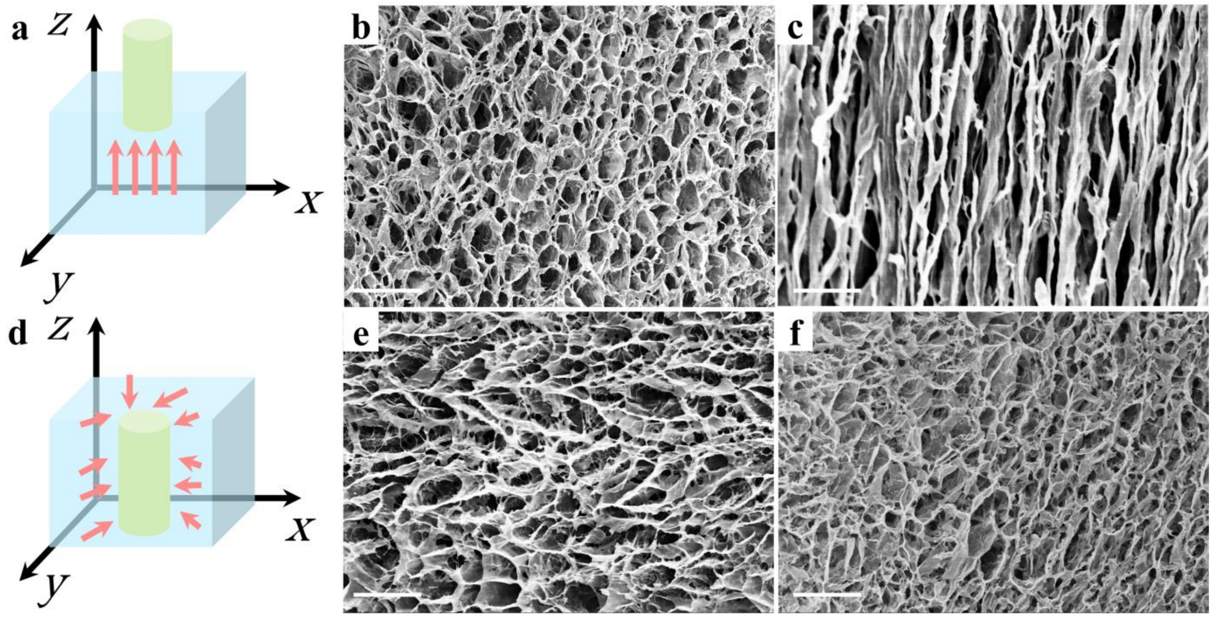

Supplementary Figure 5. a Schematic of the dipping freezing method for the PVA-AH. SEM images of the PVA-AH from the views of **b** perpendicular and **c** parallel to the *z*-axis. Scale bars: **b**, **c** 20 μm, respectively. **d** Schematic of the conventional freezing method for the PVA-IH. FESEM images of the PVA-IH from the views of **e** perpendicular and **f** parallel to the *z*-axis. Scale bars: **e**, **f** 20 μm, respectively.

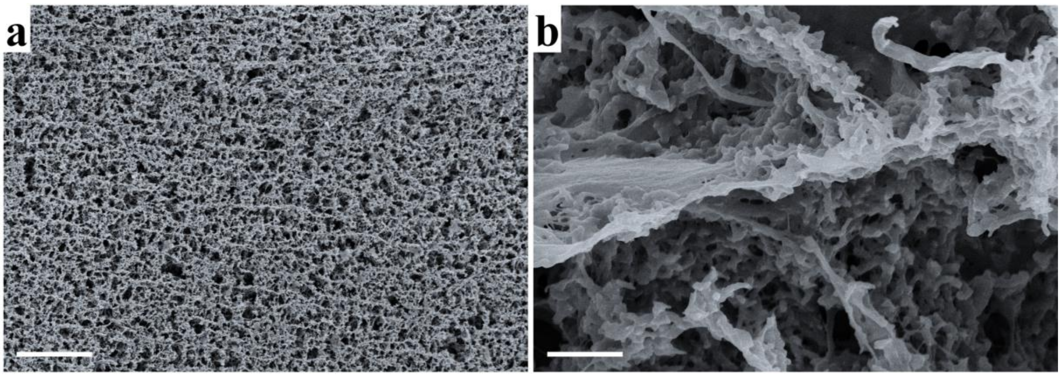

Supplementary Figure 6. SEM images of the APPH prepared with an initial aniline concentration of 1.0 M from the view of perpendicular to the *z*-axis at **a** low and **b** high magnifications, respectively. Scale bars: **a** 10 μm, **b** 1 μm.

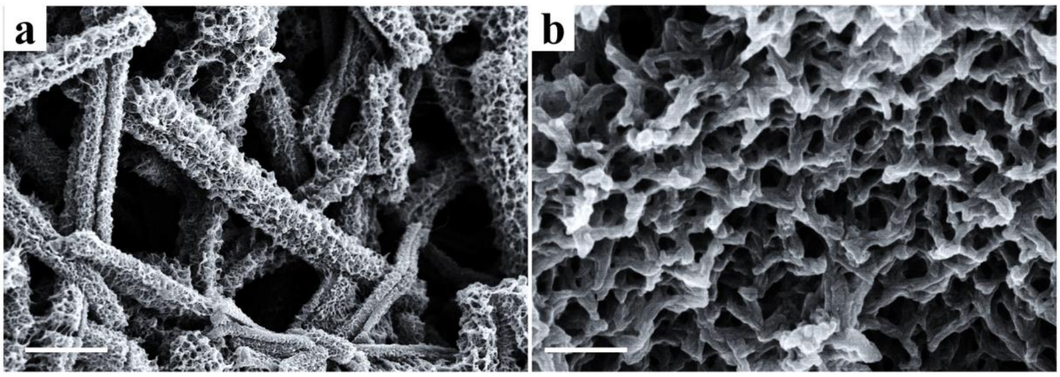

Supplementary Figure 7. SEM images of the PANI prepared by the cryopolymerization of neat aniline and APS without the addition of PVA at **a** low and **b** high magnifications, respectively. Scale bars: **a** 4 μm, **b** 200 nm.

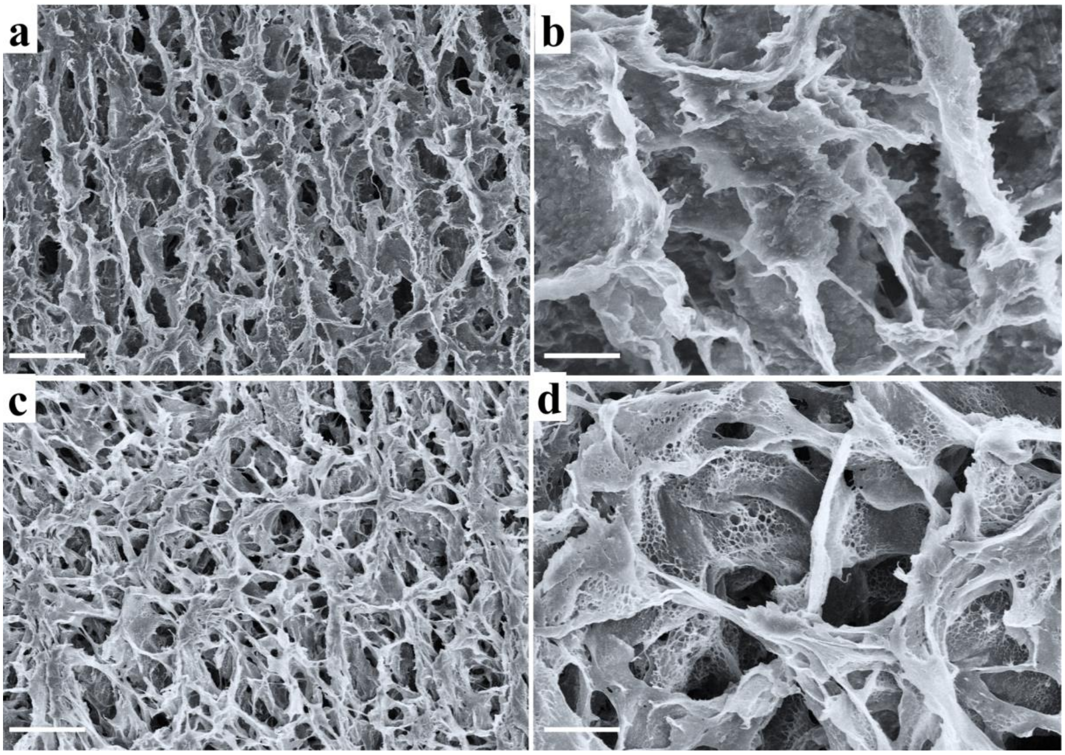

Supplementary Figure 8. SEM images of **a**, **b** CPPH and **c**, **d** IPPH. Scale bars: **a** 10 μm, **b** 2 μm, **c** 10 μm, **d** 2 μm.

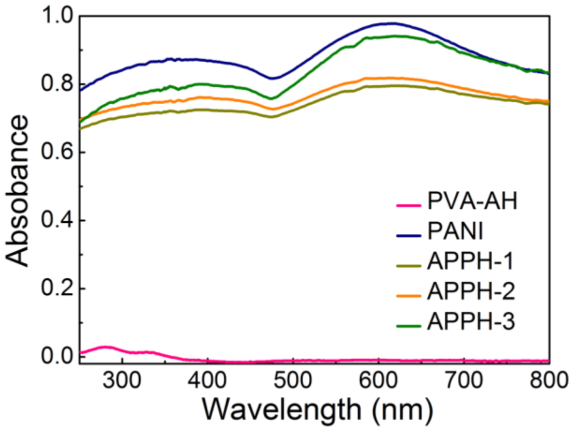

Supplementary Figure 9. UV-vis spectra of the PANI, PVA-AH and APPH.

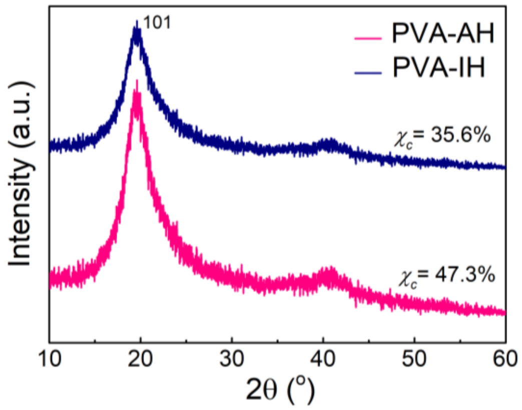

Supplementary Figure 10. XRD patterns of the PVA-AH and PVA-IH.

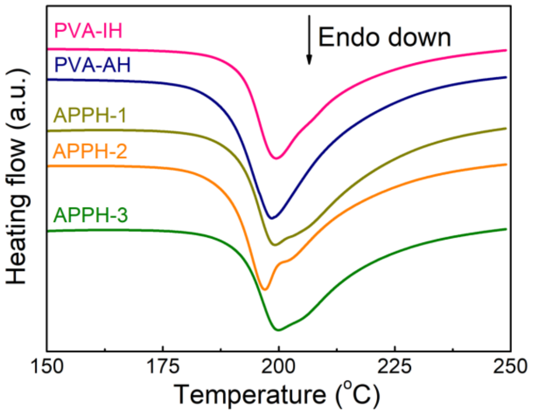

Supplementary Figure 11. DSC curves of the PVA-IH, PVA-AH and APPH samples.

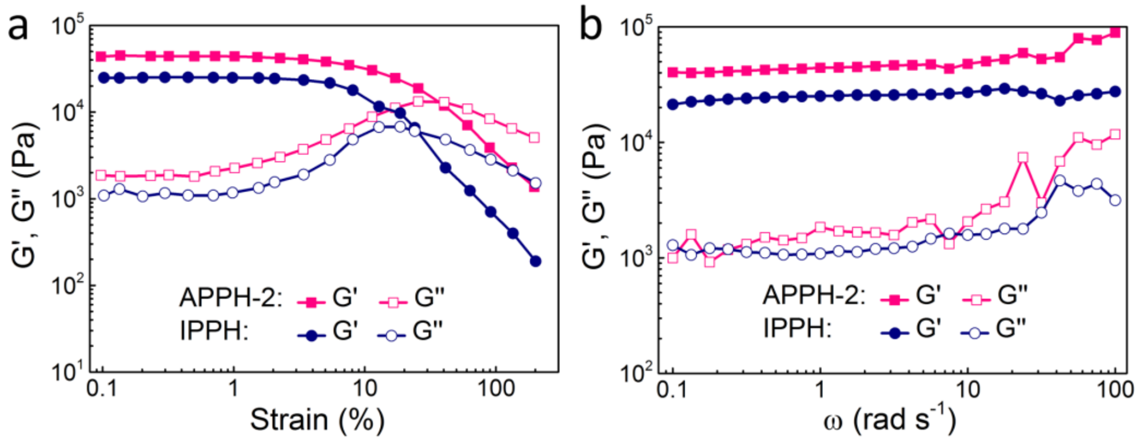

Supplementary Figure 12. Rheological properties of the APPH-2 and IPPH. **a** Strain sweeping measurements at a constant angular frequency of 1 rad s^{-1}. **b** Angular frequency sweeping measurement at a constant strain of 0.5%.

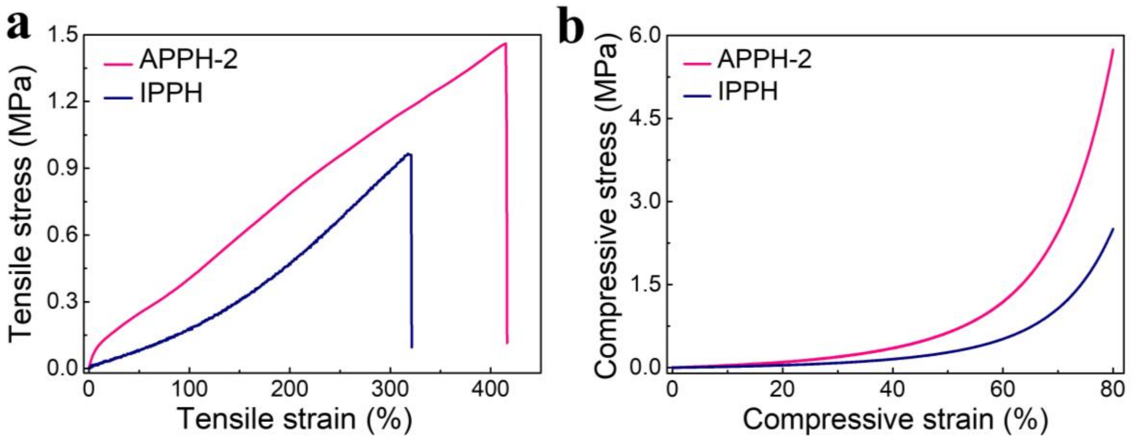

Supplementary Figure 13. a Typical tensile stress-strain curves of the APPH-2 and IPPH. **b** Typical compressive stress-strain curves of the APPH-2 and IPPH.

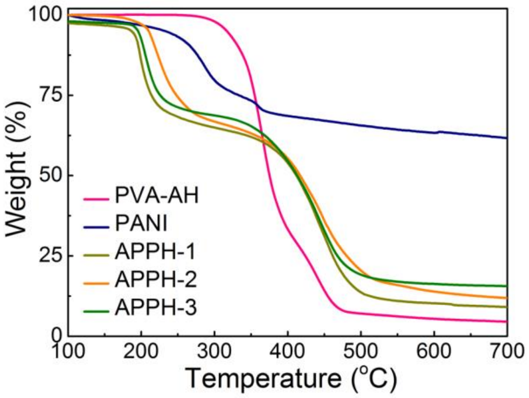

Supplementary Figure 14. TGA curves of the PVA-AH, PANI and APPH in a nitrogen atmosphere.

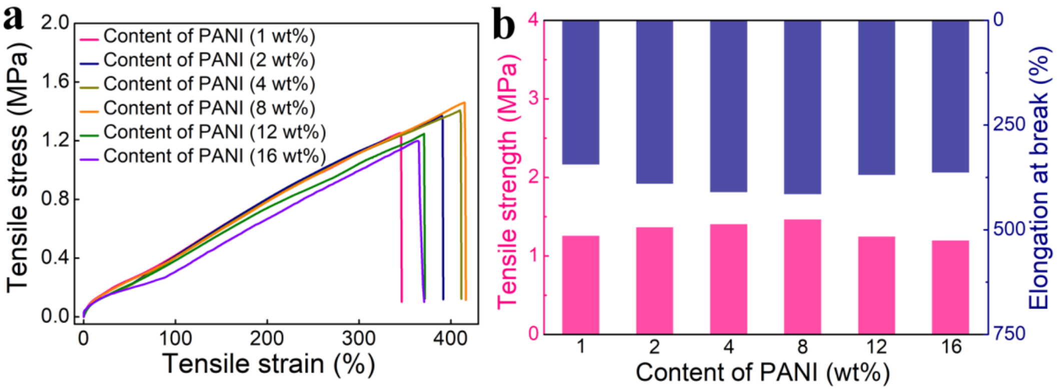

Supplementary Figure 15. a Typical tensile stress-strain curves, **b** tensile strength and elongation at break of the APPH with various contents of PANI (1, 2, 4, 8, 12, 16 wt%, respectively).

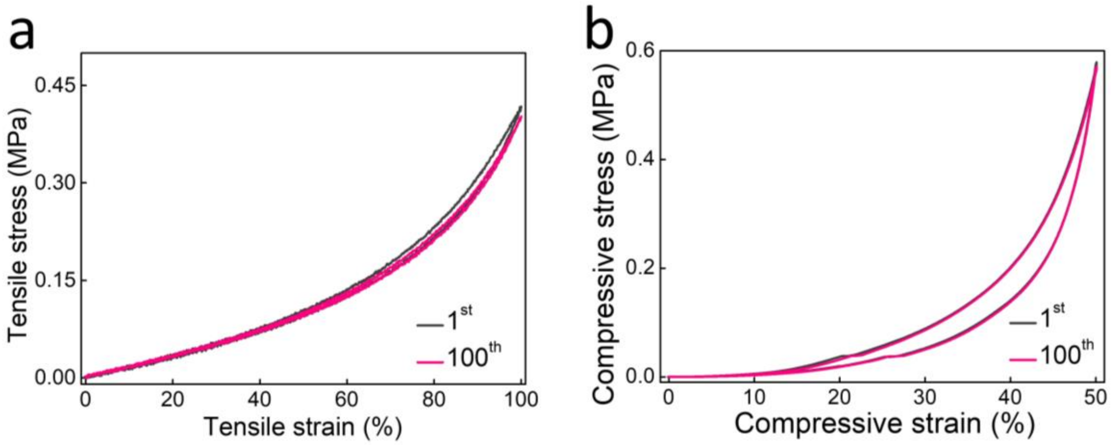

Supplementary Figure 16. a Hysteresis loops of the APPH-2 at 1st and 100th tensile-recovery cycles. **b** Hysteresis loops of the APPH-2 at 1st and 100th compression-recovery cycles.

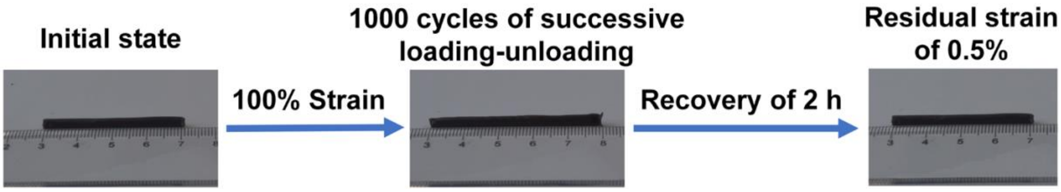

Supplementary Figure 17. Recovery properties of the APPH-2 after 1000 successive loading-unloading cycles at a tensile strain of 100%.

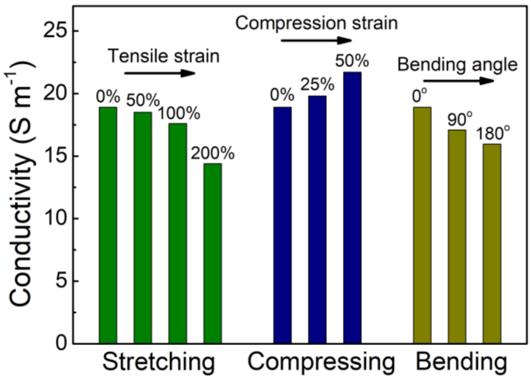

Supplementary Figure 18. Conductivity of the APPH-2 under the stretching (strain: from 0% to 200%), compression (strain: from 0% to 50%) and bending (angle: from 0° to 180°) states.

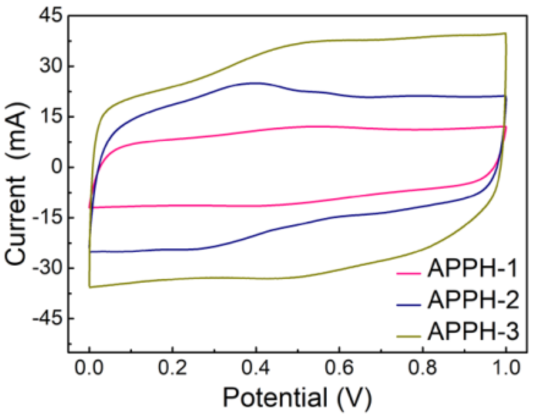

Supplementary Figure 19. CV curves of the A-SC using the APPH-1, APPH-2 and APPH-3 electrodes, respectively, at a scan rate of 10 mV s^{-1}.

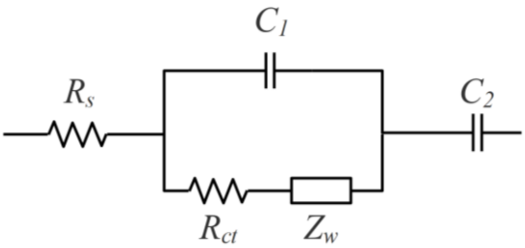

Supplementary Figure 20. The corresponding equivalent circuit diagram of the A-SC with the APPH-1, APPH-2 and APPH-3 electrodes.

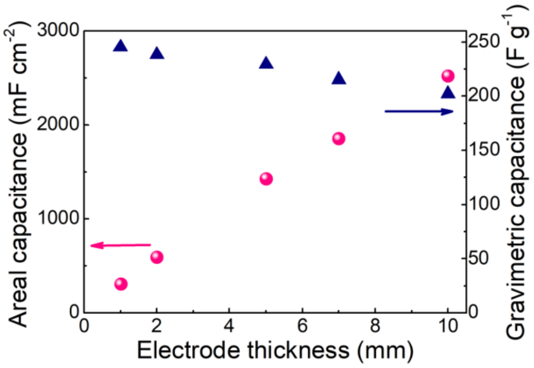

Supplementary Figure 21. Calculated gravimetric and areal capacitances of the A-SC using APPH-2 electrodes with various thicknesses, derived from the GCD curves at a current density of 1 A g^{-1}.

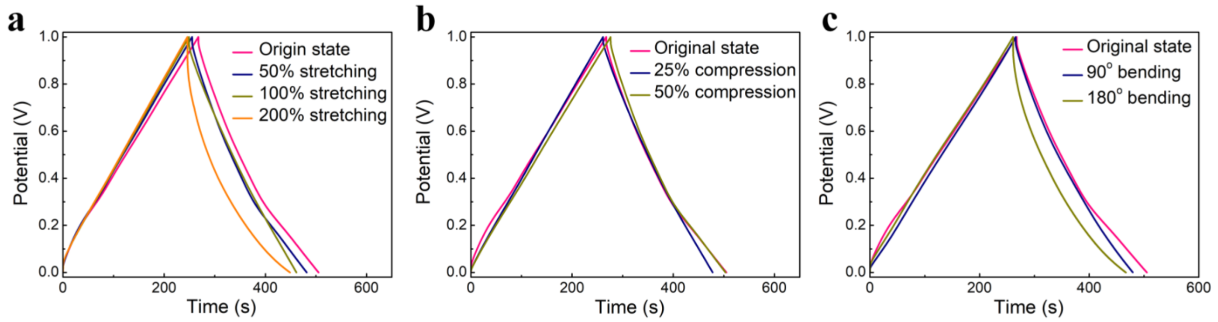

Supplementary Figure 22. GCD curves of the A-SC using APPH-2 electrodes under **a** stretching, **b** compression and **c** bending states at a current density of 1 A g^{-1}, respectively.

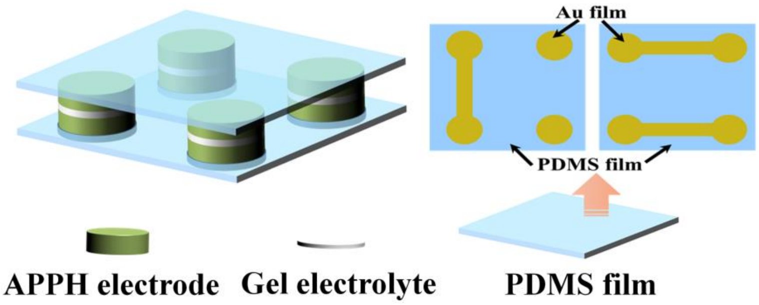

Supplementary Figure 23. Schematic illustration of the integration of four A-SC devices with a series connection by employing the PDMS substrates with Au film patterns as the conductive circuits.

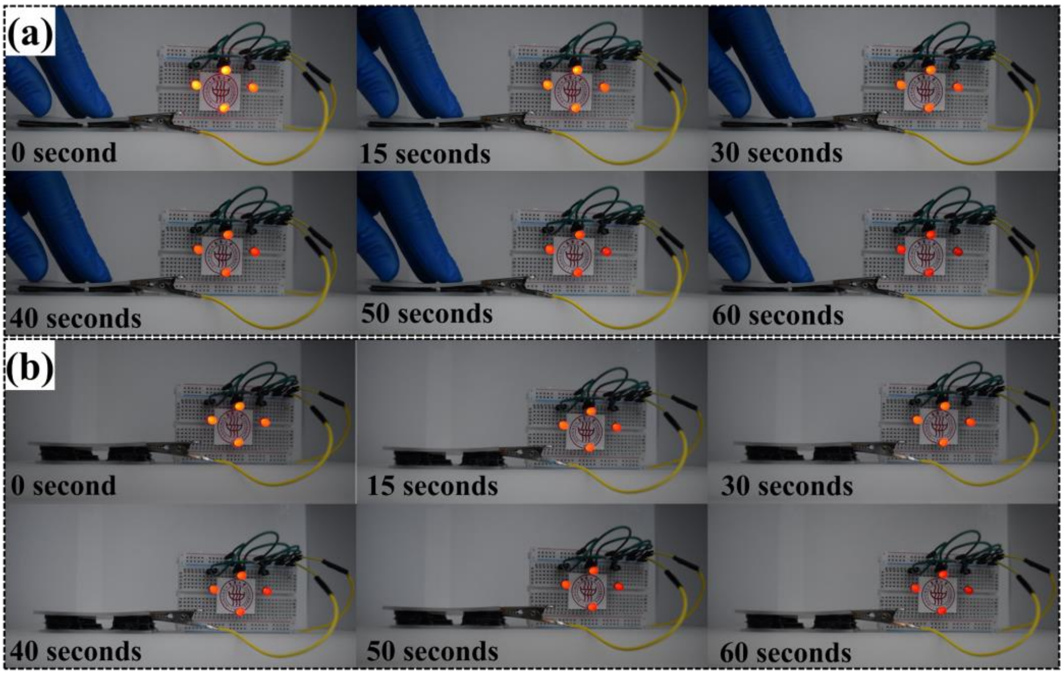

Supplementary Figure 24. Real-time photographs showing the integrated A-SC device using 4 series-connected A-SC with APPH-2 electrodes, which can lighten 4 LEDs from 0 to 60 s during the **a** compressing and **b** recovering.

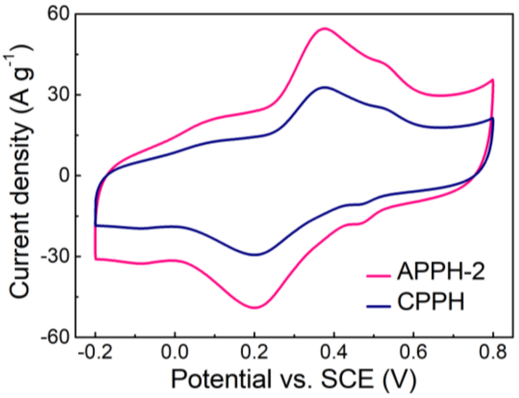

Supplementary Figure 25. CV curves of the A-SC using the APPH-2 and CPPH electrodes, at a scan rate of 50 mv s^{-1}.

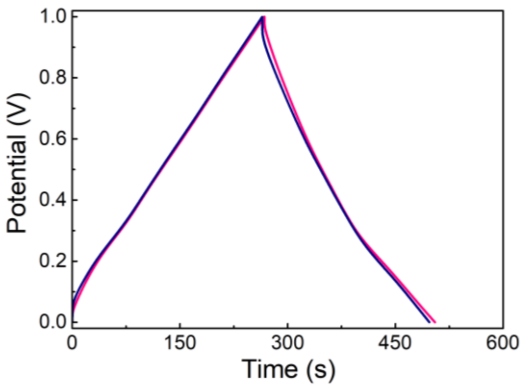

Supplementary Figure 26. GCD curves of the A-SC assembled with the current collectors (red curve) vertical and (blue curve) parallel to the vertically aligned pores among the APPH-2 electrodes, at a current density of 1 A g^{-1}.

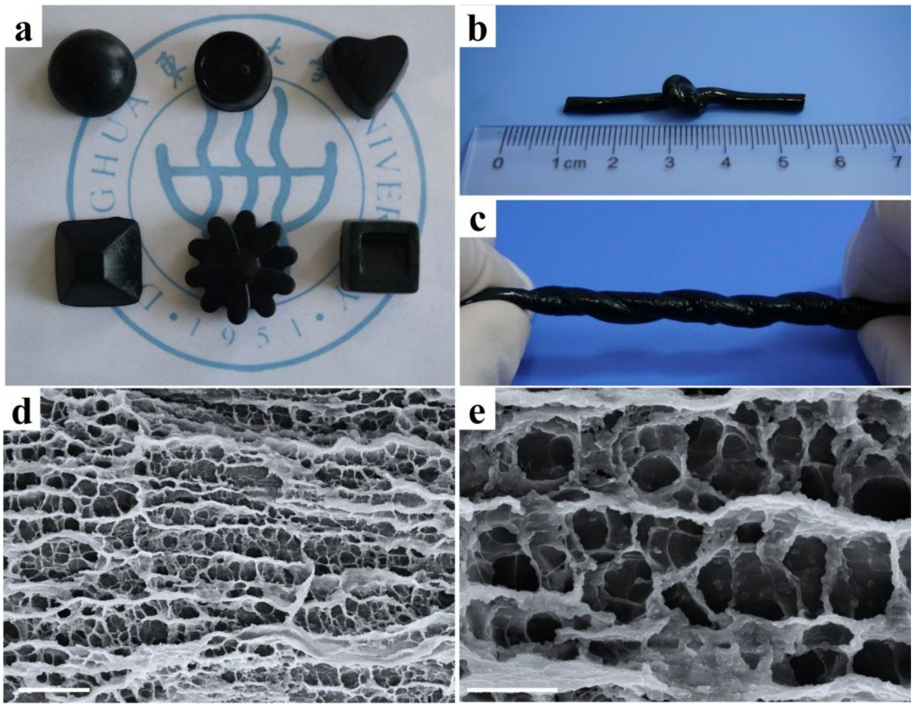

Supplementary Figure 27. a Photograph showing the diverse-shaped PVA/PPy hydrogels. Photographs showing the fiber-shaped **b** knotted and **c** twisted PVA/PPy hydrogels. **d**, **e** SEM images of the PVA/PPy hydrogels from the view parallel to the *z*-axis. Scale bars: **d** 4 μm, **e** 1 μm.

Supplementary Table 1. FTIR and Raman characteristic peaks in the anisotropic polyvinyl alcohol/polyaniline hydrogels.

FTIR spectra		Raman spectra	
Peak position (cm^{-1})	Characteristic groups	Peak position (cm^{-1})	Characteristic groups
1051	C-O stretching vibration	1170	C-H bending
1294	C-N stretching vibration	1230	C-N stretching (quinoid ring)
1491	Quinonoid ring vibration	1450	$C-N^+$ stretching (semiquinoid ring)
1572	Benzenoid ring vibration	1600	C-C deformation (semiquinoid ring)
2906	C-H stretching vibration	/	/
3224	O-H stretching vibration	/	/

Supplementary Table 2. Crystallinity (χ_c) and melting enthalpy (ΔH_m) of the polyvinyl alcohol and anisotropic polyvinyl alcohol/polyaniline hydrogels calculated from the XRD and DSC results.

Samples	χ_{c1} (%) from XRD	ΔH_m (J g^{-1})	χ_{c2} (%) from DSC
PVA-IH	35.6	77.1	39.5
PVA-AH	47.3	54.7	55.6
APPH-1	44.6	70.0	50.5
APPH-2	42.1	66.3	47.8
APPH-3	37.2	57.1	41.2

Supplementary Table 3. Elemental analysis of the freeze-dried anisotropic polyvinyl alcohol/polyaniline hydrogels.

Samples	Synthesis condition		Elemental analysis		
	Aniline (M)	PVA (wt%)	N content (wt%)	C/N mass ratio	PANI (wt%)
APPH-1	0.05	10	0.87	84.3	4.3
APPH-2	0.10	10	1.56	44.0	8.4
APPH-3	0.20	10	2.92	24.1	15.7

Supplementary Table 4. Electrochemical impedance spectroscopy data obtained from the equivalent circuit simulation of Nyquist plots in Fig. 5c by using the equivalent circuit model (Supplementary Figure 20).

Samples	R_s (Ω)	C_1 (mF)	R_{ct} (Ω)	C_2 (F)	Z_w (Ω)	Contact impedance (C_1/R_{ct})	RC time constant (s)
APPH-1	7.15	20	3.28	0.14	0.20	6.10	4.76
APPH-2	5.33	37	3.70	0.13	0.20	10.0	5.26
APPH-3	6.15	29	3.53	0.11	0.23	8.22	8.33

Supplementary Table 5. Summary of the stretchable supercapacitors using conducting polymer-based electrodes in the literature.

Electrode materials	Gravimetric capacitance of supercapacitor (F g^{-1})	Cycling stability	Mechanical performance	Refs.
PANI hydrogel	430 at 5 mV s^{-1}	85% retention after 1000 cycles	bendable	[1]
PPy hydrogel	140 at 1 A g^{-1}	90% retention after 3000 cycles	bendable	[2]
PANI grafted PVA hydrogel	153 at 1 A g^{-1}	90% retention after 1000 cycles	bendable	[3]
PANI-GO hydrogel	112 at 0.08 A g^{-1}	86% retention after 17000 cycles	bendable	[4]
PVA-PANI hydrogel	210 at 0.25 A g^{-1}	107% retention after 2000 cycles	foldable	[5]
SWCNT/PANI foam	216 at 0.64 A g^{-1}	92% retention after 1000 cycles	compressible	[6]
APPH-2	260 at 0.5 A g^{-1}	90% retention after 2000 cycles	stretchable, compressible, bendable	This work

Supplementary Table 6. Electrochemical impedance spectroscopy data obtained from the equivalent circuit simulation of Nyquist plots in Fig. 6d by using the equivalent circuit model (Supplementary Figure 20).

Sample states	R_s (Ω)	C_1 (mF)	R_{ct} (Ω)	C_2 (F)	Z_w (Ω)	Contact impedance (C_1/R_{ct})	RC time constant (s)
Original	5.33	37	3.70	0.13	0.20	10.0	5.26
200% stretching	9.74	15	3.16	0.11	0.24	4.75	11.2
50% compressing	5.46	33	2.93	0.14	0.18	11.26	4.76
180° bending	9.14	16	3.27	0.11	0.20	4.89	7.69

Supplementary References

1. Shi, Y. et al. Nanostructured conductive polypyrrole hydrogels as high-performance, flexible supercapacitor electrodes. *J. Mater. Chem. A* **2**, 6086-6091 (2014).
2. Wang, K. et al. Flexible solid-state supercapacitors based on a conducting polymer hydrogel with enhanced electrochemical performance. *J. Mater. Chem. A* **2**, 19726-19732 (2014).
3. Li, W., Gao, F., Wang, X., Zhang, N. & Ma, M. Strong and robust polyaniline-based supramolecular hydrogels for flexible supercapacitors. *Angew. Chem. Int. Ed.* **128**, 9342-9347 (2016).
4. Li, P. et al. Stretchable all-gel-state fiber-shaped supercapacitors enabled by macromolecularly interconnected 3D graphene/nanostructured conductive polymer hydrogels. *Adv. Mater.* **30,** 1800124 (2018).
5. Li, W., Lu, H., Zhang, N. & Ma, M. Enhancing the properties of conductive polymer hydrogels by freeze-thaw cycles for high-performance flexible supercapacitors. *ACS Appl. Mater. Interfaces* **9**, 20142-20149 (2017).
6. Niu, Z., Zhou, W., Chen, X., Chen, J. & Xie, S. Highly compressible and all-solid-state supercapacitors based on nanostructured composite sponge. *Adv. Mater.* **27**, 6002-6008 (2015).
