# Supplementary material for: Cryopolymerization enables anisotropic polyaniline hybrid hydrogels with superelasticity and highly deformation-tolerant electrochemical energy storage
Source: Nat Commun. 2020 Jan 7;11:62. doi: 10.1038/s41467-019-13959-9 (PMC6946679; doi:10.1038/s41467-019-13959-9)
Supplement: Supplementary file 2 — Description of additional supplementary files [file 41467_2019_13959_MOESM2_ESM.docx]

Description of additional supplementary files

Title: Supplementary Movie 1

Description: Cyclic stretching tests of the anisotropic polyvinyl alcohol/polyaniline hydrogel at a 100% strain.

Title: Supplementary Movie 2

Description: Cyclic compression tests of the anisotropic polyvinyl alcohol/polyaniline hydrogel at a 50% strain.
